# Will obesity break your heart - cardiac biomarkers in the Japan Morning Surge-Home Blood Pressure study

**DOI:** 10.1038/s41440-023-01560-z

**Published:** 2024-01-10

**Authors:** Jan-Niklas Hönemann, Jens Jordan

**Affiliations:** 1 Department of Internal Medicine III, Division of Cardiology, Pneumology, Angiology, and Intensive Care, University of Cologne, Cologne, Germany; 2 Institute of Aerospace Medicine, German Aerospace Center (DLR), Cologne, Germany; 3 Medical Faculty, University of Cologne, Cologne, Germany

**Keywords:** Obesity, Cardiovascular risk, Hypertension, Biomarker

## Abstract

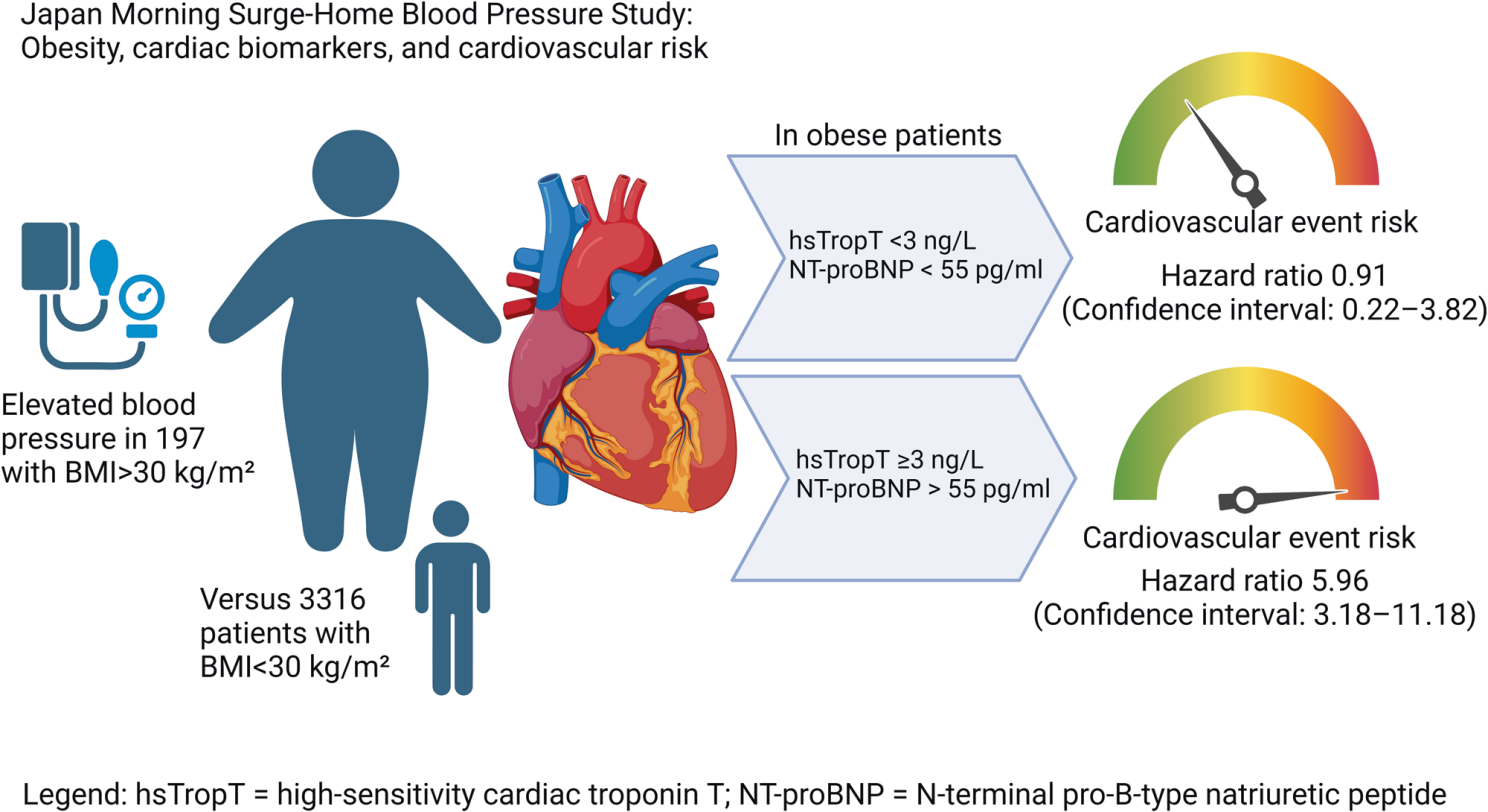

Over the last decades, the prevalence of obesity massively increased in many countries [1]. The subsequent increase in obesity-associated diseases will impose a burden on affected patients, health care systems, and societies alike. Patients with obesity are more likely to have arterial hypertension, require more medications to have their blood pressure controlled, and are overrepresented among patients with treatment-resistant arterial hypertension. Moreover, obesity is associated with additional cardiovascular and metabolic risks, particularly an increased risk of experiencing type 2 diabetes mellitus. Therefore, we will have to counsel and clinically manage more patients with obesity and arterial hypertension in years to come. Current hypertension guidelines have begun to provide guidance regarding risk assessment and treatment of patients with obesity and arterial hypertension; however, the clinical trials evidence supporting these recommendations is still emerging [2].

As new treatment options, such as more potent weight loss medications, enter clinical practice, cardiovascular and metabolic risk stratification for the patient in front of us will be crucial. However, traditional risk factors are not sufficient to gauge overall cardiometabolic risk. Moreover, the relationship between adiposity and metabolic or cardiovascular risk shows large inter-individual variability. For example, some patients with obesity do not show perturbed glucose and lipid metabolism, a phenomenon sometimes referred to as metabolic healthy obesity [3]. The state-of-affairs is further complicated by the epidemiological observation that increased adiposity can also be associated with decreased morbidity and mortality in various diseases including heart failure, the so-called “obesity paradox” [4]. Yet, a recent clinical trial showed that patients with heart failure and preserved ejection fraction benefit from pharmacological weight loss through treatment with the glucagon-like peptide 1 receptor agonist (GLP1-RA) semaglutide [5]. Overall, risk prediction in patients with obesity is by no means trivial. Vascular biomarkers hold promise in refining cardiovascular risk assessment of patients with obesity [6]. However, these measurements can be time consuming, require additional equipment, and are, therefore, not widely available.

The study by Watanabe et al. [7] in this issue suggests that risk prediction in Japanese patients with arterial hypertension and obesity can be improved through circulating cardiovascular biomarkers that are easily determined in venous blood samples. The authors assessed N-terminal pro B-type Natriuretic Peptide (NTproBNP) and high-sensitivity Troponin T, which are both established clinical biomarkers markers in cardiovascular medicine. In the acute setting, troponins are useful markers of myocardial cell necrosis while NTproBNP, which responds to myocardial stretch, is widely applied in heart failure management. The recent European Society of Hypertension guidelines advocate NTproBNP and high-sensititvity Troponin measurements in a more chronic setting when assessing hypertension-mediated organ damage [2]. The study by Watanabe et al. included 3,513 participants of the Japan Morning Surge-Home Blood Pressure (J-HOP) with arterial hypertension. The authors stratified patients according to body mass index into a normal weight group (<25 kg/m²), an overweight group (25–29.9 kg/m²), and an obese group (≥30 kg/m²). Then, they divided body mass index groups in into groups with lower and with higher circulating NTproBNP (cutoff 55 pg/mL) and high-sensitivity Troponin T (cutoff 3 ng/mL). The authors assessed independent and combined associations of body mass index and each circulating biomarker or both biomarkers combined on cardiovascular events. The primary outcome of interest was cardiovascular disease incidence comprising fatal and nonfatal coronary artery disease, fatal and nonfatal stroke, and fatal and hospitalized heart failure.

Over a mean follow-up period of 6.4 years, the authors ascertained 232 cardiovascular events, namely 113 coronary events, 79 strokes, and 40 heart failure events. After adjusting for age and sex, patients with obesity exhibited an approximately threefold increased risk of experiencing a cardiovascular event. NTproBNP and high-sensitivity Troponin measurements provided additional information, particularly in patients with obesity. In fact, an increase in both biomarkers identified a group of patients with obesity at strikingly high cardiovascular risk.

An important limitation of the study is that groups differed in terms of important confounding variables such as age, prescription of antihypertensive drugs, type 2 diabetes mellitus prevalence, high density lipoprotein-cholesterol, estimated glomerular filtration rate, and blood pressure. While the study suggests that NTproBNP and high-sensitivity troponin T predicts risk in patients with obesity and arterial hypertension, interpretation of these biomarkers is not always straightforward. For example, obesity is associated with a paradoxical reduction in circulating natriuretic peptides, which may contribute to metabolic disease. However, natriuretic peptides appear to increase as cardiovascular disease emerges [8]. Moreover, a recent study in participants of the Tromsø 7 study in Northern Norway suggested that NTproBNP and high-sensitivity Troponin T measurements appear to have low sensitivities when screening for heart failure in the general population [9].

Despite these issues, Watanabe et al. [7] should be commended for providing an important piece of evidence that may aid in managing patients with arterial hypertension and obesity in the future. Perhaps, such measurements could be applied to target treatments to patients with obesity most likely to benefit. In addition to refining antihypertensive and lipid lowering therapies, the approach could be utilized when prescribing weight loss interventions. While healthy eating and physical exercise can be recommended for most people, they rarely help to attain sustained reductions in body weight in patients with obesity. Over many years, weight loss medications exhibited limited efficacy but were fraught with side effects. A new generation of drugs shows substantially improved efficacy and safety profiles. For example, once weekly treatment with the semaglutide decreased body weight by 15.8% compared 6.4% on daily liraglutide treatment [10]. Over 72 weeks, once weekly treatment with tirzepatide, a dual glucose- dependent insulinotropic polypeptide and GLP-1 RA, reduced body weight dose-dependently by up to 20.9% [11]. These treatments will be costly and health benefits may vary between patients. Bariatric surgery is another approach to elicit substantial weight loss. In addition to sustained weight loss, bariatric surgery can improve cardiovascular and metabolic morbidity and mortality. However, these benefits have to be weighed against operative risks, the need for long-term nutritional counselling, and substantial costs [12]. Perhaps, circulating biomarkers such as those studied by Watanabe et al. [7] may help to give better advice to our patients with obesity and arterial hypertension. Implementing all this knowledge in clinical practice will be challenging. We dare to speculate that artificial intelligence-based approaches may help us to cope with this complexity, allow for a more individualized treatment approach, and save time that is better spent talking to our patients [13].
